# The genome of *Candidatus phytoplasma* ziziphi provides insights into their biological characteristics

**DOI:** 10.1186/s12870-023-04243-6

**Published:** 2023-05-12

**Authors:** Chaoling Xue, Yao Zhang, Hongtai Li, Zhiguo Liu, Weilin Gao, Mengjun Liu, Huibin Wang, Ping Liu, Jin Zhao

**Affiliations:** 1 College of Life Science, Hebei Agricultural University, Baoding, 071000 China; 2 Key Laboratory of Hebei Province for Plant Physiology and Molecular Pathology, Hebei Agricultural University, Baoding, 071000 China; 3 Research Center of Chinese Jujube, Hebei Agricultural University, Baoding, 071000 China

**Keywords:** Jujube, Phytoplasma, Biological characteristics, Codon usage bias, Metabolic pathways, Mn-SodA, FtsHs

## Abstract

Phytoplasmas are obligate cell wall-less prokaryotic bacteria that primarily multiply in plant phloem tissue. Jujube witches’ broom (JWB) associated with phytoplasma is a destructive disease of jujube (*Ziziphus jujuba* Mill.). Here we report the complete ‘*Candidatus Phytoplasma* ziziphi’ chromosome of strain Hebei-2018, which is a circular genome of 764,108-base pairs with 735 predicted CDS. Notably, extra 19,825 bp (from 621,995 to 641,819 bp) compared to the previously reported one complements the genes involved in glycolysis, such as *pdhA*, *pdhB*, *pdhC*, *pdhD*, *ackA*, *pduL* and *LDH*. The synonymous codon usage bias (CUB) patterns by using comparative genomics analysis among the 9 phytoplasmas were similar for most codons. The ENc-GC3s analysis among the 9 phytoplasmas showed a greater effect under the selection on the CUBs of phytoplasmas genes than mutation and other factors. The genome exhibited a strongly reduced ability in metabolic synthesis, while the genes encoding transporter systems were well developed. The genes involved in sec-dependent protein translocation system were also identified.The expressions of nine FtsHs encoding membrane associated ATP-dependent Zn proteases and Mn-SodA with redox capacity in the *Ca. P.* ziziphi was positively correlated with the phytoplasma concentration. Taken together, the genome will not only expand the number of phytoplasma species and provide some new information about *Ca. P.* ziziphi, but also contribute to exploring its pathogenic mechanism.

**Supplementary Information:**

The online version contains supplementary material available at 10.1186/s12870-023-04243-6.

## Introduction

Phytoplasmas are cell wall-less phytopathogenic bacteria [1] and the diseases associated with phytoplasmas have posed a serious threat to several hundred economically important plants production [2]. They are naturally transmitted by phloem-feeding insects of vectors, mainly leafhoppers (Cicadellidae), plant hoppers (Fulgoroidea) and psyllids (Psyllidae) [1]. Lacking of axenic phytoplasma culture is contributed for the phytoplasmas biological character progress lagged in comparison to cultivable bacteria [3]. However, high throughput genome sequencing was adopted as a powerful tool to characterize these bacteria [4–7]. It will further facilitate the comparative genomics analysis among the phytoplasma species. To date, more than 30 phytoplasma completed or drafted genomes have been published [4–27] (Supplementary file-Table S1), and these data indicate that they have small genomes lacking many metabolic pathways. Based on the latest progress in phytoplasma taxonomy, the genetically diverse phytoplasma have been classified into 37 groups from three aspects (nomenclature, classification, identification) [3, 28]. According to homology comparative analysis, the lower conservation among phytoplasma genomes indicated their huge diversity at the genome level [29]. Hence, the new phytoplasma genome will expand the number of phytoplasma species.

Jujube witches’ broom (JWB) in jujube (*Ziziphus jujuba* Mill.) is associated with subgroup 16SrV-B ‘*Candidatus Phytoplasma* ziziphi’ (‘*Ca. P.* ziziphi’) [30]. Jujube is widely grown in Asia and represents one of the most economically important fruit trees for achieving poverty alleviation in arid and semiarid areas. JWB is a typical disease associated with phytoplasma, the diseased trees exhibit a variety of symptoms, such as small and etiolated leaves, phyllody and witches’ broom. And the diseased trees can’t bear fruit and then died within 2–3 years [31]. Moreover, most jujube cultivars are sensitive to *Ca. P.* ziziphi, thus the disease exhibits highly infectious in jujube orchards. *Ca. P.* ziziphi mainly transmit through insect vectors, such as *Hishimonus sellatus* Uhler, *Hishimonoides aurifacialis* Kuoh and *Typhlocyba sp*. Germar [32, 33]. The large-scale destruction by JWB has caused huge economic and ecological losses in jujube production. At present, trunk injection with antibiotic is the main solution to cure JWB-diseased trees [33], but the solution can’t prevent the infection of new healthy trees. Thus, the genome sequencing of *Ca. P.* ziziphi would provide many valid clues for exploiting their weaknesses to prevent their invasion into host plant.

During jujube-phytoplasma interaction process, reactive oxygen species (ROS) were produced in jujube tissues and then the antioxidant defense system was obviously triggered to protect the jujube cell from the ROS damage [34]. In general, superoxide dismutase (SOD) plays an important role in ROS degradation [35]. However, it is unclear whether *Sod* gene exists in *Ca. P.* ziziphi and if it can function in protecting the cells of *Ca. P.* ziziphi from ROS damage.

Proteins secreted via the Sec system are likely to be important during the infection process. The SecA, SecY and SecE, as the essential components of Sec protein translocation system, have been identified in onion yellows phytoplasma (OY) [36] and they are required for protein translocation and cell viability in *Escherichia coli*[37]. Some secreted proteins in phytoplasma, such as SAP54, SAP11, TENGU, SAP05 and Zaofeng 6 were reported [9, 38–41]. However, the components of Sec protein translocation system in *Ca. P.* ziziphi remain to be determined.

Here we used next-generation DNA sequencing technology to sequence and assemble the genome of *Ca. P.* ziziphi, and the genome obtained was larger than the previous one [29]. The pattern of codon usage bias (CUB) among 9 phytoplasma genomes was compared, and the basic metabolic pathways and transporter systems of *Ca. P.* ziziphi were analyzed. Meantime, some specific genes such as *SodA*, sec-dependent translocation system genes and *FtsH* were also identified in *Ca. P.* ziziphi.

## Results

### Genome assembly, annotation and characterization of ***Ca. P.*** ziziphi

**Fig. 1:**
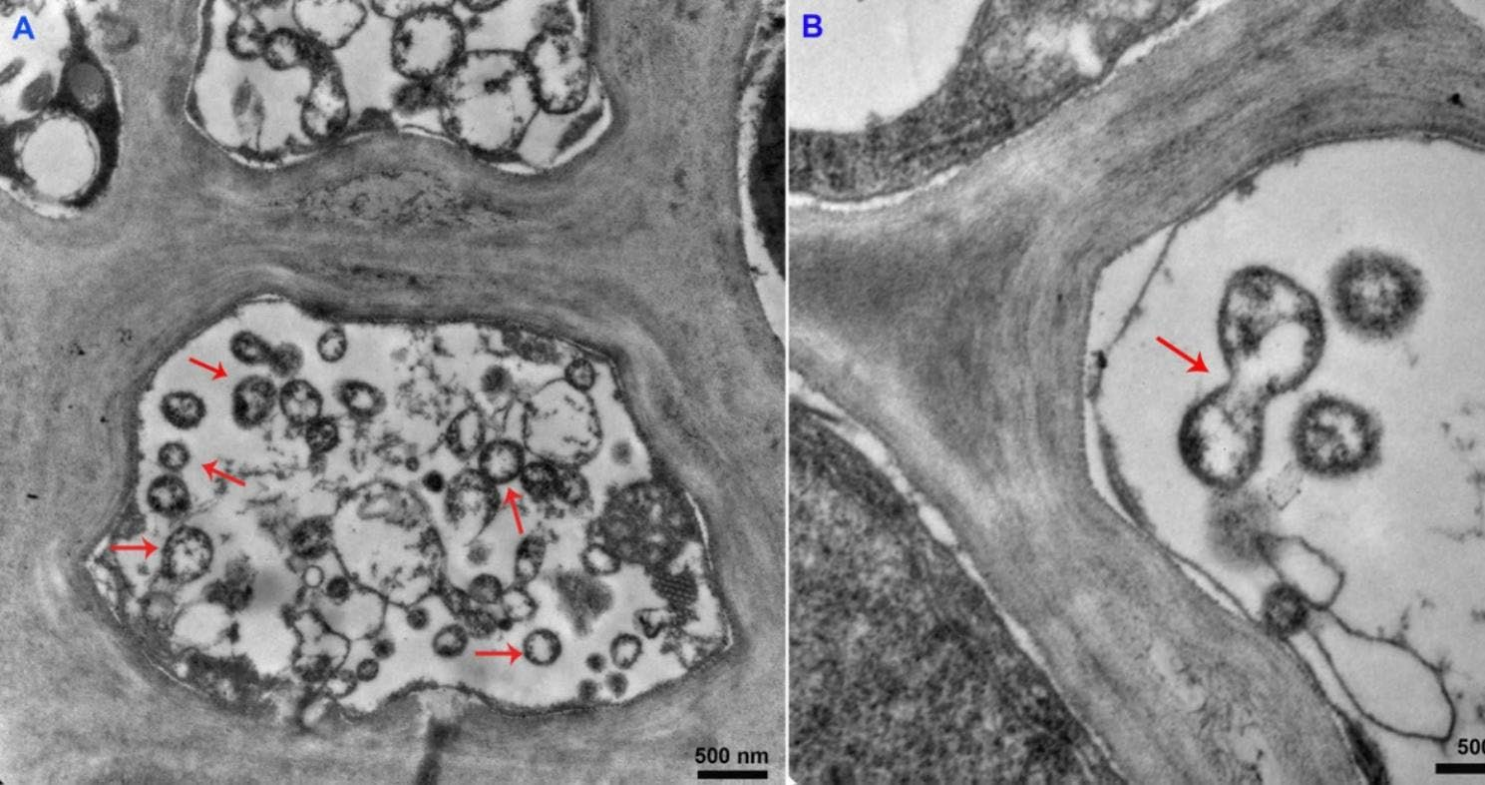
The detection of *Ca. P.* ziziphi by transmission electron microscopy (TEM). **(A)** Phytoplasmas in the cytoplasm of sieve elements (SEs). **(B)** Phytoplasmas reproduce by splitting one cell into two. The red arrow indicates phytoplasmas

**Fig. 2:**
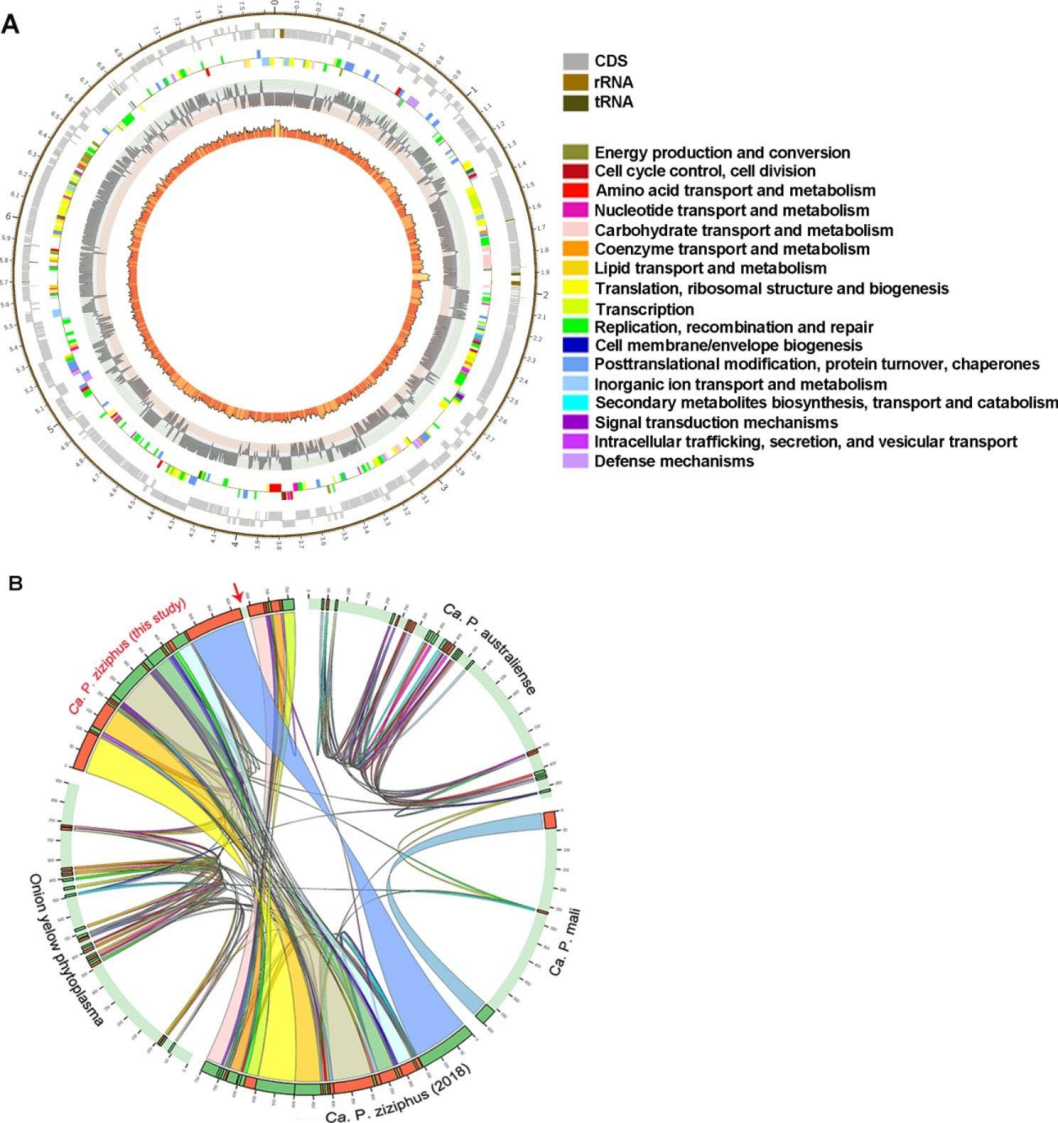
Characteristics of the genome of *Ca. P.* ziziphi and gene synteny analysis with other phytoplasmas. **(A)** Map of the genome of *Ca. P.* ziziphi. The rings from the outside to the inside are as follows: ring 1 shows the scale in kb; ring 2 shows the locations of CDS, rRNA genes and tRNA genes; ring 3 shows the COG to which each CDS belongs; ring 4 shows the GC-skew value ((G-C)/(G + C)); ring 5 shows the content of GC. **(B)** Whole-genome alignment reveals gene synteny. The chromosomes are shown in circle form to illustrate relative gene synteny at *Ca. P.* ziziphi with other three phytoplasmas. The red arrow shows the 19-kb sequence, which location is range from 621 995 bp to 641 819 bp in the new genome of *Ca. P.* ziziphi

Phytoplasmas are growing in the sieve elements (SEs) of diseased tissues. In diseased jujube tissues, large numbers of *Ca. P.* ziziphi were observed in SEs, and they were generally round in shape and about ~ 300 nm in diameter (Fig. 1A). *Ca. P.* ziziphi proliferated through cell division (Fig. 1B). Its genome consists of one chromosome of 764, 108 bp (Fig. 2A), and its chromosome is a circular DNA molecule with 23.22% G + C content. The genome contains 735 open reading frames (ORFs), comprising 76.16% of the genome; 701 protein coding genes with assigned functions, two operons for rRNA genes and 32 tRNA genes were annotated.

Compared to the previously reported genome (Wang et al., 2018) [29], this assembly contains 19.825 kb (starting from 621,995 to 641,819 bp) of additional sequence (Fig. 2B), which was verified by PCR amplification in the linking regions between the new fragment and previously reported fragment (Supplementary file-Fig. S1). 7 genes (*pdhA, pdhB, pdhC, pdhD, ackA, pduL, LDH*) involved in glycolysis metabolism were annotated in the new fragment. The gene synteny analyses showed that the arrangements and inversions existed in the phytoplasma genomes (Fig. 2B).

### Codon usage among nine Phytoplasmas from different host plant species

**Fig. 3:**
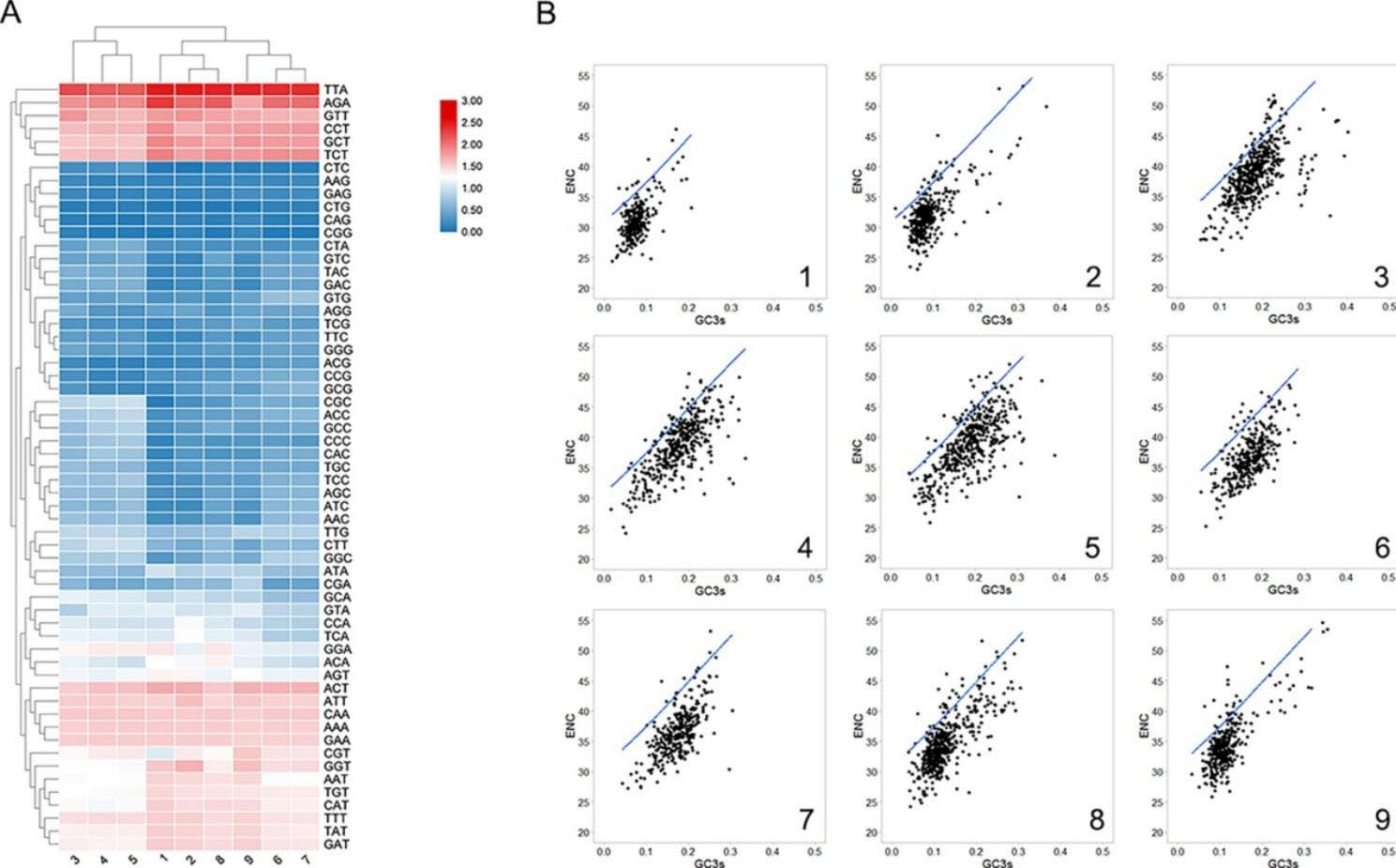
Codon usage bias and amino acid composition among 9 phytoplasma species. **(A)** Based on the RSCU values of all 59 synonymous codons, a heat map was constructed by biclustering to describe the variations in CUB patterns among 9 phytoplasmas. **(B)** ENc-plot curves were used to explore the influence of GC3s on the CUB patterns of 9 phytoplasmas. 1-*Ca. P.* oryzae GCA_001578535.1; 2-*Ca. P.* mali GCA_000026205.1; 3- (Ca. P. australiense) Strawberry lethal yellows phytoplasma (CPA) GCA_00039 7185.1; 4- (*Ca. P.* asteris) Aster yellows witches’-broom phytoplasma GCA_000012225.1; 5- (*Ca. P.* asteris) Onion yellows phytoplasma GCA_000009845.1; 6 (*Ca. P.* vaccinium)Vaccinium witches’-broom phytoplasma GCA_ 000309405.1; 7- (*Ca. P.* italian) Italian clover phyllody phytoplasma GCA_000300695.1; 8- (*Ca. P.* ziziphi) JWB phytoplasma (in this study); 9- (*Ca. P.* aurantifolia) Peanut witches’-broom phytoplasma GCA_000364425.1

To elucidate the pattern of synonymous codon usage bias (CUB) among phytoplasma genomes, the relative synonymous codon usage (RSCU) values of all 59 codons of *Ca. P.* ziziphi and other 8 phytoplasmas were compared and analyzed. The CUB patterns among the 9 phytoplasmas were similar for most codons (Fig. 3A), which provided significant insights pertaining to their classification and evolution. Overall, the CUB patterns were more similar in the relative species (*Ca. P.* ziziphi, *Ca. P.* mali and *Ca. P.* oryzae), (*Ca. P.* australiense (CPA), *Ca. P.* asteris (AY-W and OY-W) and (*Ca. P.* aurantifolia (PnWB), *Ca. P.* italian and *Ca. P.* vaccinium). As shown in Fig. 3A, the classification based on the CUB pattern agreed with the previous study [42], in which AY-W and OY-W, *Ca. P.* Italian and *Ca. P.* vaccinium belonged to the 16Sr I and 16Sr III groups, respectively.

The expected effective number of codons (ENc) plot, an effective tool to describe codon usage patterns, was used to explore the influence of GC3s on CUBs among 9 phytoplasmas (Fig. 3B). Based on the previous studies [43–48], mutation is the main force shaping codon usage when the genes points fall near the expected curve. While the genes points fall considerably below the expected curve, selection is the main force shaping codon usage. As shown in Fig. 3B, most genes of 9 phytoplasmas were located below the expected ENc-plot curve while only a few points lay lie in close proximity, indicating that natural selection played the major role in the codon usage bias of phytoplasma genomes, however, the effect of the mutation pressure and other factors (the gene length and the base composition) on codon usage bias was slight and also irreplaceable.

In this study, the tendency of 9 phytoplasma to use less G + C for all codon positions was consistent with previous study [49]. Thus, this leaded to more TTA instead of CTN to encode Leucine and more AGA instead of CGG to encode Arginine. Three high-frequency codons (TTA, AGA, TCT), as well as low-frequency codons (CAG, CTG, CGG) were also identified in the genome of *Ca. P.* ziziphi. The top six most frequent codons were the same among the 9 genomes (Supplementary file-Table S2) and their amino acid compositions were similar (Supplementary file-Fig. S2).

### The basic metabolic pathways in ***Ca. P.*** ziziphi

**Fig. 4:**
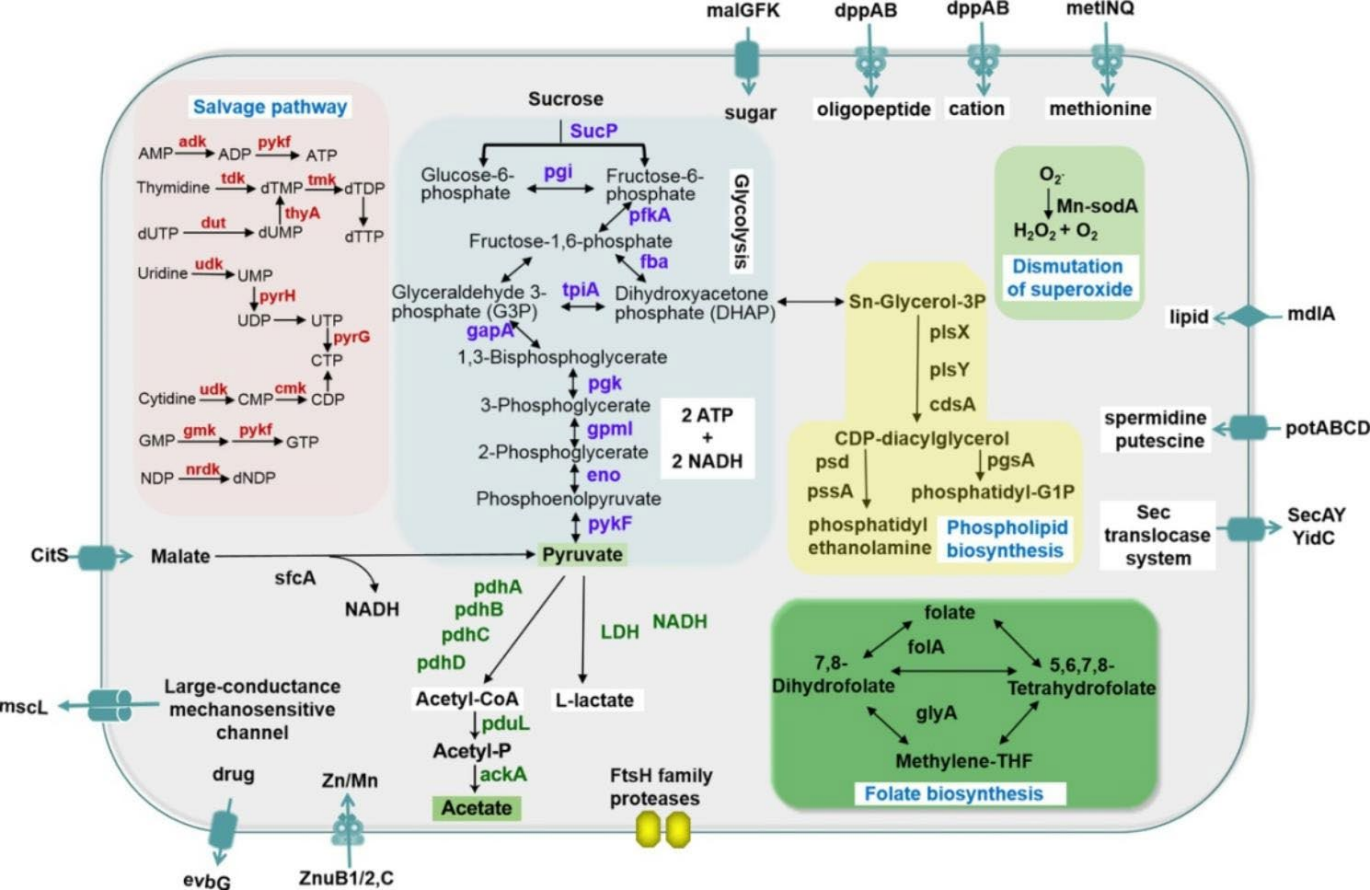
An overview of the metabolic pathways in *Ca. P.* ziziphi. Based on the set of genes with predicted functions, the main metabolic pathways and various transporters are shown. The red color letters represent the genes related to salvage pathway of synthesizing nucleotides. The blue and green color letters represent the complete glycolysis pathway synthesizing NADPH and ATP in the genome of *Ca. P.* ziziphi. In which the genes associated with pyruvate to acetate pathway were missed in the previous genome (Wang J et al., 2018) and were represented in green color letters. The yellow box displays the pathway of phospholipid biosynthesis. The dark green box represents the pathway of folate biosynthesis. The light green box shows the pathway of dismutation of superoxide. The genes of various and developed transport systems, such as citS, sfcA, MalKFG, dppAB, potABCD, ZnuB1/2,C, metINQ, display on the cell membrane

Similar to other phytoplasmas, the metabolic pathways in the *Ca. P.* ziziphi genome lack genes encoding F1F0 ATP synthase and the pentose phosphate cycle (Fig. 4). In parallel, the genes related to the salvage pathway (*adk*, *cmk*, *dut*, *gmk*, *hit*, *nrdE*, *nrdF*, *pyrG*, *pyrH*, *tdk*, *thyA*, and *tmk*) play the function as synthesizing nucleotides in *Ca. P.* ziziphi (Fig. 4). The genes involved in glycolysis (*pgi*, *pfkA*, *fba*, *tpiA*, *gapA*, *pgk*, *gpmI*, *eno*, *pykF*) were found to replace the genes in the pentose phosphate cycle. Moreover, some missing genes in the previous genome [29]were annotated in this study, including four enzymes (*pdhA*, *pdhB*, *pdhC*, *pdhD*) involved in the pyruvate to acetyl-CoA pathway and an enzyme (*ackA*) using acetyl-p and ADP as substrates to produce acetate and ATP. Phosphate propanoyltransferase (*pduL*), which can catalyze acetyl-CoA as a substrate to produce acetyl-p, was annotated. The Lactate dehydrogenase (*LDH*) involved in the formation of NADH from pyruvate to lactate was also identified. Therefore, the complete glycolysis pathway synthesizing NADPH and ATP was presented in this genome.

For assimilating and transporting metabolites from host cells, the developed transport systems including 35 genes were discovered in the genome of *Ca. P.* ziziphi. The malate/ citrate symporter (*citS*) and malate dehydrogenase (*sfcA*) were found. To import sugars from the host environment, the components of the ABC transporter for maltose (MalKFG) were presented. Other transporters involved in dipeptide/oligopeptide (dppAB), spermidine/ putrescine (potABCD), Mn/Zn (ZnuB1/2, C) and methionine transport (metINQ) were also annotated. Meantime, the genes involved in folate (*folA* and *glyA*) and phospholipid biosynthesis (*plsX, plsY, cdsA, psd, pssA, pgsA*) and multidrug resistance (*SodA*, *evbG* and *mdlA*) were also identified, suggesting that they are required for basic metabolism in *Ca. P.* ziziphi and contribute to high adaptability to distinct and complex host environments.

### The Sec-dependent protein translocation system in ***Ca. P.*** ziziphi

**Fig. 5:**
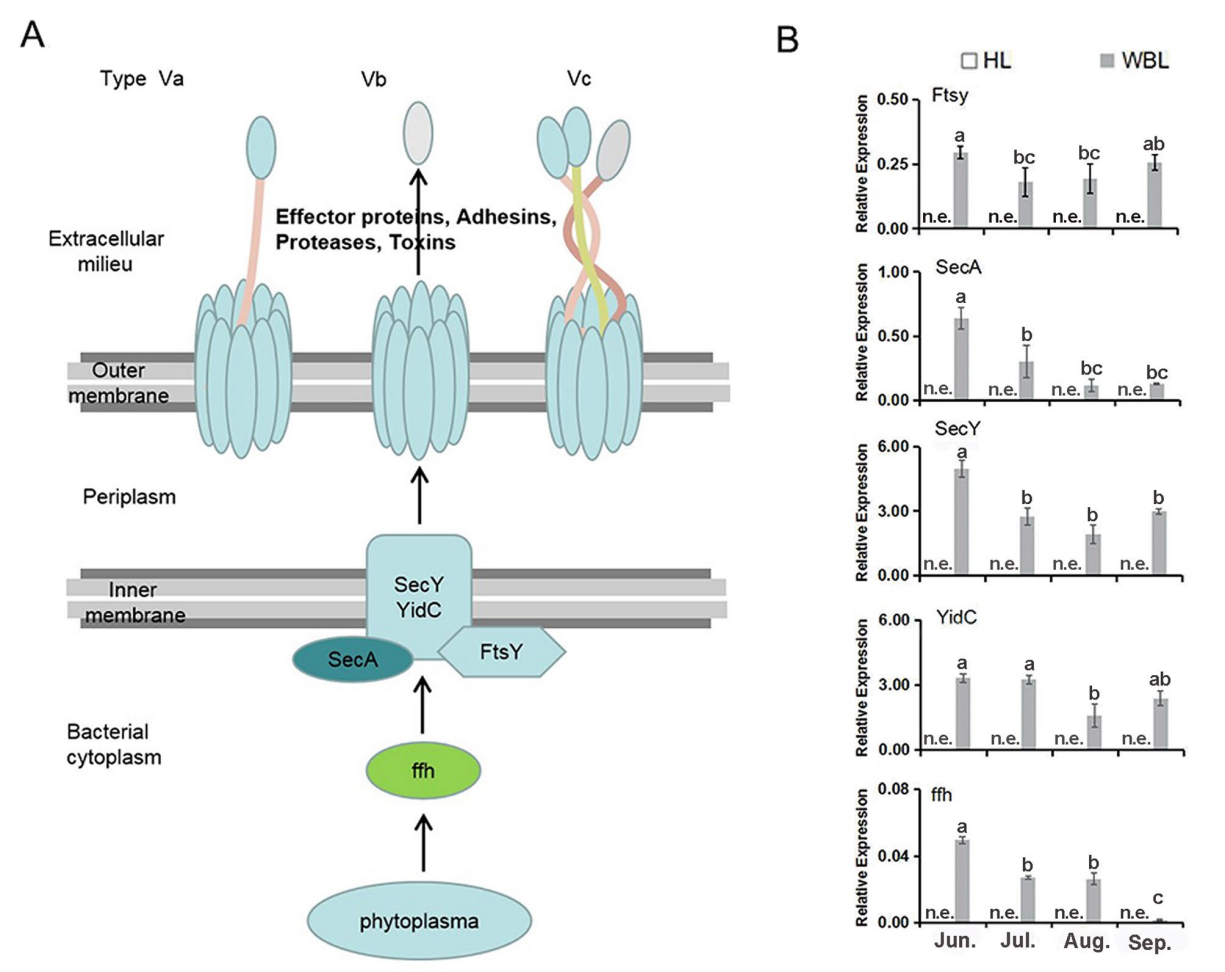
The sec-dependent protein translocation system is identified in *Ca. P.* ziziphi. **(A)** The components of the Sec-pathway in *Ca. P.* ziziphi. A two-step Type Vb secretory pathway dependent on Sec is presented. The secreted proteins were first recognized by ffh and then bound to its receptor Ftsy. With the help of SecA, SecY and YidC, the secreted effector would successively transport in the periplasm and extracellular environment across the membrane [51–53]. **(B)** The expression of genes involved in the Sec pathway in healthy (HL) and diseased witches’ broom leaves (WBL). The data are mean ± SD of three biological replicates. Different letters between bars indicate significant differences at p < 0.05 (Duncan’s multiple range test). n.e., no expression

Sec-dependent pathway including three components (a motor protein, a membrane integrated conducting channel and the SecYEG translocase) is considered one of the most highly conserved protein secretion pathways ubiquitous in all domains of bacteria life [50, 54]. The effectors secreted by phytoplasmas can directly transit into host cells via Sec-dependent pathway [55]. The genes related to Sec-dependent protein translocation system including *ffh*, *Ftsy*, *SecA*, *SecY* and *YidC* were identified in *Ca. P.* ziziphi (Fig. 5A). However, *secE* was not observed in the genome but in some other phytoplasmas (*Ca. P.* tritici (WBD), *Ca. P.* asteris (OY-M, AY-WB), *Ca. P.* australiense, *Ca. P.* mali) [9, 10], the lacking component of Sec-dependent system in *Ca. P.* ziziphi suggested that the diversity and conservative exited among 16 S-groups phytoplasmas.

According to the bacteria secreted system [50], secretory pathway with highly efficient protein secretion mechanism in *Ca. P.* ziziphi was presented (Fig. 5A) [51–53], in which secreted proteins were first recognized by *ffh* and then bound to its receptor Ftsy. With the help of *SecA*, *SecY* and *YidC*, the secreted effectors should successively transport in the periplasm and extracellular environment across the membrane.

Furthermore, the expression of *ffh*, *Ftsy*, *SecA,SecY* and *YidC* was measured in diseased leaves and showed a similar trend at different infection periods (Fig. 5B). The cooperation of these components of the secretion system should be required for *Ca. P.* ziziphi to transport a variety of proteins, such as virulence factors, proteases and toxins.

### Mn-type SodA in ***Ca. P.*** ziziphi owned redox activity

**Fig. 6:**
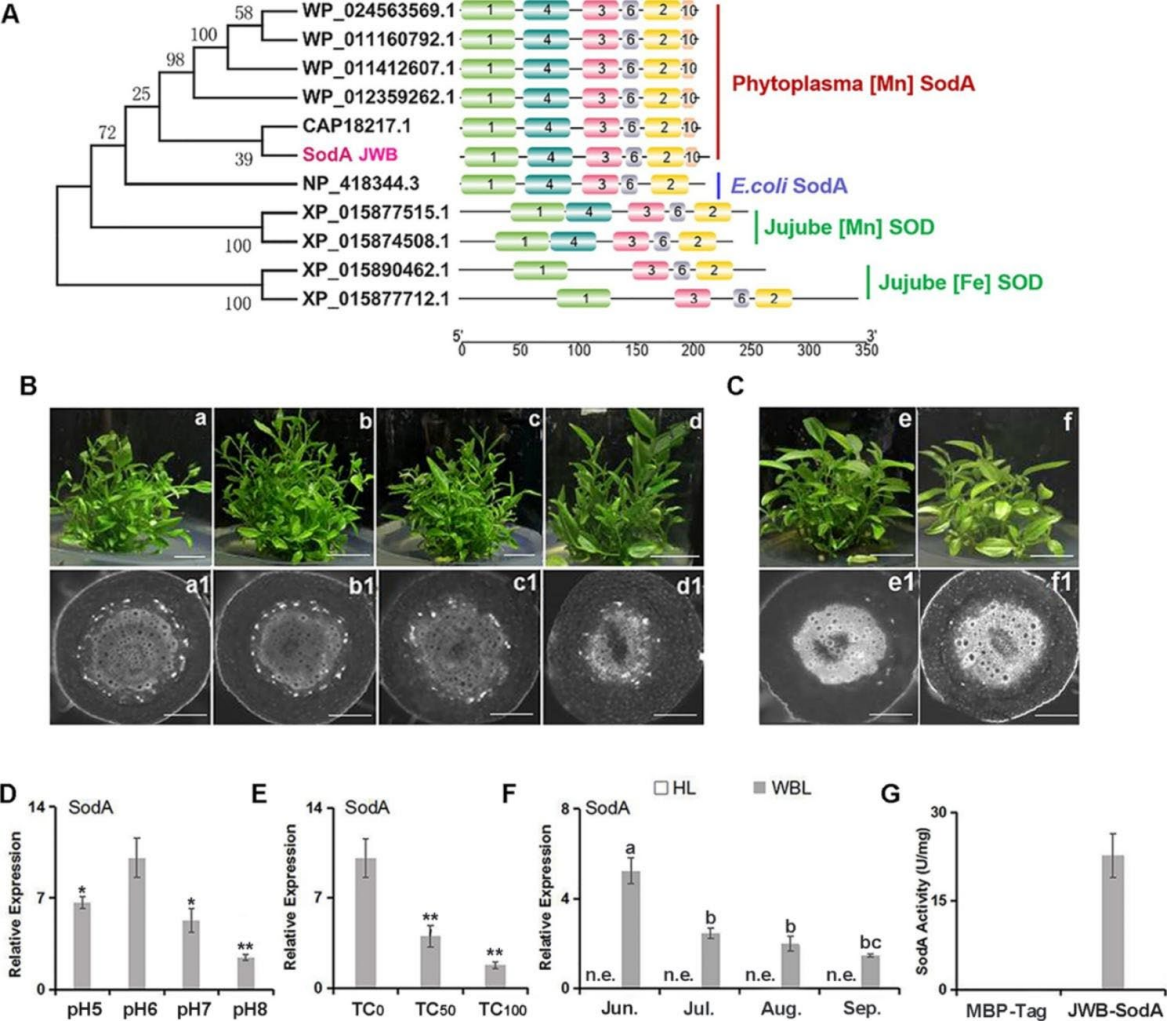
Phylogenetic tree construction, expression and enzymatic activity determination of Mn-SodA in *Ca. P.* ziziphi. **(A)** Phylogenetic tree and motif analysis of SodA in phytoplasmas (WP_024563569.1 Wheat blue dwarf phytoplasma (*Ca. P.* tritici); WP_011160792.1 Chrysanthemum yellows phytoplasma (*Ca. P.* asteris); WP_011412607.1 Aster yellows witches’-broom phytoplasma (*Ca. P.* asteris); WP_012359262.1 *Ca. P.* australiense; CAP18217.1 *Ca. P.* mali.), E. coli (NP_418344.3 Escherichia coli str. K-12 substr. MG1655) and jujube SODs (XP_015877515.1 ZjSOD [Mn], mitochondrial; XP_015874508.1 ZjSOD [Mn] mitochondrial-like; XP_015890462.1 ZjSOD [Fe] 3, chloroplastic; XP_015877712.1 ZjSOD [Fe], chloroplastic-like). (B, C) Morphological phenotypes (a, b, c, d, e, f) and phytoplasmas detected by DAPI (a1, b1, c1, d1, e1, f1) in JWB plantlets under treatments of pH (B) and tetracycline (C). (D, E) The expression of Mn-SodA in JWB plantlets under pH (D) and tetracycline treatments (E). More white fluorescence spots in sieve elements illustrate more phytoplasmas in diseased tissues. JWB plantlets cultured in media with pH 6 were used as controls. TC0, TC50 and TC100 represent JWB plantlets treated with 0 µg/mL, 50 µg/mL and 100 µg/mL tetracycline, respectively. Bar = 2 cm (a, b, c, d, e, f); Bar = 100 μm (a1, b1, c1, d1, e1, f1). Asterisks represents significant differences from the control plantlets (cultured in media with pH 6 (D) or plantlets treated with TC0 (E)) at *p < 0.05, **p < 0.01 (Student’s *t*-test). **(F)** SodA expression was detected in diseased jujube leaves. Different letters between bars indicate significant differences at p < 0.05 (Duncan’s multiple range test). **(G)** The enzyme activity of Mn-SodA in *Ca. P.* ziziphi by NBT assay. MBP-Tag protein was used as a negative control. n.e., no expression

Only one SOD protein encoded by Mn-type *SodA* was found in the genome of *Ca. P.* ziziphi, and then its function was further investigated. The phylogenetic tree showed that SodA proteins in phytoplasmas were highly homologous to Mn-dependent ZjSODs in jujube (Fig. 6A, Supplementary file-Fig. S3), indicating that the sequences and functions of Mn-dependent SODs are more conserved than that of Fe-dependent SODs during evolution. Based on the motif analysis, Motif 4 and Motif 10 in SodAs of phytoplasmas were Mn- dependent and unique domains, respectively (Supplementary file-Fig. S3).

To elucidate the relationship between the expression of SodA and the content of *Ca. P.* ziziphi, a series of regulatory treatments were implemented in JWB plantlets (Fig. 6B and C). The treatments with higher pH (7 and 8) and tetracycline (50 µg/mL and 100 µg/mL) to host jujube plantlets could inhibit the reproduction of *Ca. P.* ziziphi (Supplementary file-Fig. S4), and the expressions of SodA in *Ca. P.* ziziphi were also significantly decreased (Fig. 6D and E). Compared to other periods, the highest expression of SodA in *Ca. P.* ziziphi was observed in June, i.e. the early stage of infection (Fig. 6F). The results showed that the expression pattern of SodA was consistent with the content change of *Ca. P.* ziziphi.

Furthermore, to determine whether the SodA in *Ca. P.* ziziphi possess enzyme activity, the recombinant MBP-SodA proteins were purified from BL21 strain using amylose agarose and its enzyme activity was verified based on inhibition of nitroblue tetrazolium (NBT) reduction (Fig. 6G), indicating that SodA can function in the redox capacity.

### FtsHs were identified in ***Ca. P.*** ziziphi

**Fig. 7:**
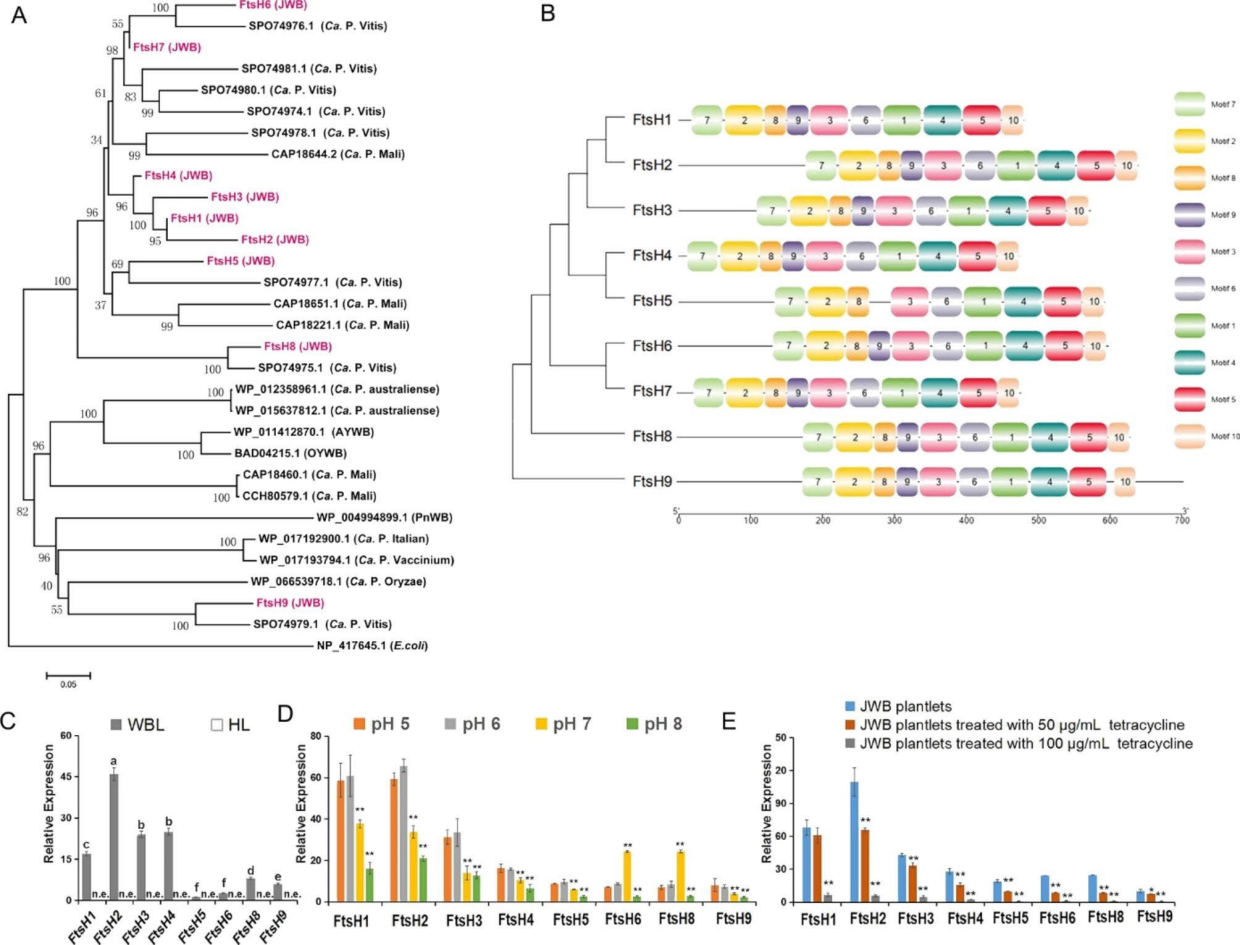
Phylogenetic tree construction, conserved motifs and expression analyses of FtsHs in *Ca. P.* ziziphi. **(A)** The phylogenetic tree of FtsHs in JWB and other phytoplasmas (FtsH of E. coli (NP_417645.1) was used as an out-group control). The NJ tree was constructed from the amino acid sequences of FtsHs using MEGA 11 with 1000 bootstrap replicates. **(B)** Conserved motifs of the FtsHs arranged according to their phylogenetic relationships. Ten conserved motifs were identified using Multiple Em for Motif Elicitation (MEME) and are shown in different colors. **(C, D, E)** The expression of FtsH genes was detected in diseased and healthy tissues **(C)** and in JWB plantlets treated with different pH values **(D)** and tetracycline concentrations **(E)**. The treatments of JWB plantlets were the same as in Fig. 6F and G. The data are mean ± SD of three biological replicates. Error bars represent the standard deviation. Asterisks indicate significant difference at **p < 0.01 (Student’s *t*-test). Different letters between bars indicate significant differences at p < 0.05 (Duncan’s multiple range test). And n.e., no expression

The FtsHs (hflB gene) encoding membrane-associated ATP-dependent Zn proteases are anchored in the cell membrane [56]. Nine copies of FtsHs were identified in the genomeof *Ca. P.* ziziphi (Supplementary file-Table S3). The amino acid (aa) length of the FtsH genes ranged from 440 (FtsH4) to 665 (FtsH9).Phylogenetic analysis showed that the number and phylogenetic location of FtsHs in *Ca. P.* ziziphi was extremely similar with that in *Ca. P.* vitis (Fig. 7A), indicating that their functions may be conservative.

The domain and sequence motif analysis showed that FtsHs in *Ca. P.* ziziphi were highly conserved (Fig. 7B and Supplementary file-Fig. S5) and contained a typical AAA module of the AAA family. The AAA module contained the characteristic sequence motifs, namely Walker A (motif 2) and Walker B (motif 8) as well as the pore residues (motif 2) and the second region of homology (SRH) (motif 3) fingerprint. The ‘zinc-binding’ motif (motif 1, HEAGH)was identified function as protease active centre. Other motifs (4, 5, 6, 7, 9, 10) were secondary structural elements that formed the protease domain.

All of the FtsH genes tested were expressed in diseased leaves, and some of them showed high expression (Fig. 7C). In the JWB plantlets under the treatments with pH and tetracycline, their expression was positively correlated with the content of *Ca. P.* ziziphi (Fig. 7D and E).

## Discussion

CUB is an essential feature of most genomes and can be influenced by nucleotide distribution, the GC content, translational selection, protein hydropathy and protein secondary structure, tRNA and aminoacyl tRNA synthase genes set, tRNA modification systems [54–61]. And the correlation analysis of CUB between the host and pathogen might also provide insights into better understanding their interactions. Here, the CUB patterns of the genomes of jujube and *Ca. P.* ziziphi were compared, and the results showed that the CUB patterns of some codons were similar and that their top six most frequent codons encoded the same amino acids (Supplementary file-Table S4). Moreover, similar results were observed in the other 2 phytoplasmas and their host plants (Supplementary file-Table S4). Generally, there are large differences in the compositions of amino acids among various genomes [62]. In mycoplasma, their synonymous codon usage pattern obviously differs from that of their natural host [63]. It needs to study that whether the similar usage patterns of most codons between phytoplasmas and host plants is helpful to their survival in the host environment.

The phytoplasmas are propagated in sieve elements, which are nutrient-rich environments. The extend genome data produced in this study constitutes a useful resource for investigating its pathogenicity as well as their survival strategies in host cells. In the whole, their genome adapts to the habitat life by reductive evolution, i.e., reducing many essential metabolisms. To save energy and raw materials, the salvage pathway replacing *de novo* synthesis was chosen by *Ca. P.* ziziphi to synthesize nucleotides. The incomplete nucleotide synthesis pathway also existed in *Rickettsia prowazekii* [64] with a similar endocellular lifestyle. Thus, the reductive evolution might be a useful strategy of *Ca. P.* ziziphi to survive in host plant.

None of the ATP synthase subunits or ATP/ADP translocases was found in the genomeof *Ca. P.* ziziphi, which is an important gene set for life organisms. Thus, phytoplasma is the organism to be discovered that can survive without these gene sets. From the genome analysis, ATP synthesis in *Ca. P.* ziziphi is strongly dependent on the glycolysis pathway. The pentose phosphate cycle also synthesizes NADPH and thus reconstitutes the bacterium redox homeostasis. The presence of the two genes (citS and sfcA) and the absence of the pentose phosphate cycle mean that *Ca. P.* ziziphi can use malate to produce pyruvate and promote the synthesis of NADH and ATP, and the uptake and utilization of citrate and malate may provide the main sources for carbohydrate metabolism [65].

A large number of genes encoding transporter systems present in the genome of *Ca. P.* ziziphi are conducive to aggressively import metabolites from host jujube sap, which is rich in nutrition. For phytoplasmas, their consumption of metabolites from host cells obviously disturbed the metabolic balance of the host plant, such as photosynthetic, carbohydrate and hormone metabolism [31, 34, 66]. To satisfy the consumption of phytoplasma and itself, jujube trees must enhance various metabolic pathways after phytoplasma invasion [31]. Thus, the heavy metabolite absorption of phytoplasma from host cells results in the overburden, exhaustion and eventual death of jujube trees.

Compared to healthy leaves, the Mn content in diseased jujube leaves was obviously and gradually decreased according to the developmental periods [67]. In this study, the expression of *Ca. P.* ziziphi Mn-SodA was also gradually reduced with the prolongation of infection periods. Taken together, the lower Mn content in host jujube was agreed with the decreased expression of *SodA* in *Ca. P.* ziziphi, indicating that the *SodA* function might depend on Mn content.

Multicopy FtsH proteins are conserved among bacteria and involved in protein secretion and membrane protein assembly as well as adaptations to nutritional conditions and osmotic stress [68, 69]. Most bacteria, such as *Escherichia coli* and *Bacillus* subtilis, have only one *FtsH* [5, 70], while seven *FtsH*s were identified in the AY phytoplasma strains (OY and AY-WB) [7]and nine *FtsHs* were found in the genome of *Ca. P.* ziziphi. To the small-scale phytoplasma genomes, such a large number of FtsHs is a significant feature. In *Staphylococcus aureus*, the mutation of FtsH could lead to attenuated pathogenicity [71]. In *B. subtilis*, FtsH is involved in endospore development by degrading Spo0E and SpoVM [69–74], suggesting that FtsH may participate in cell division and reproduction. In Synechocystis, FtsH regulates the levels of GgpS [75], which is necessary for the synthesis of the osmoprotectant glucosylglycerol. The expression of FtsHs in *Ca. P.* ziziphi was obviously modulated by different pH stresses (Fig. 7), suggesting that it is sensitive to the changes in acid-base homeostasis. To avoid harmful accumulation, FtsHs also can function in the degradation of subunit SecY [68, 76]. Overall, the versatility of the FtsHs in phytoplasmas is important for their adaptability to the host environment, which might explain why phytoplasmas require more of these proteins. Whether FtsHs in phytoplasma are involved in the secretion of pathogenic factors and the detailed regulatory mechanism need further research.

## Conclusions

Phytoplasmas cause diseases in several hundred economically plants worldwide, and jujube witches’ broom disease (JWB) is a typical and destructive phytoplasma disease. There are few studies concerning their survival strategies in host environment. Here, we obtain the complete JWB phytoplasma genome and present evidence on how is codon usage selected in phytoplasma genomes during evolution in host cells. Through integrating genome, transcriptional level and metabolic data, we focused on clarifying the basic metabolic characterization of phytoplasma genome and analyzing some curial genes that was positively correlated with the phytoplasma concentration in host plant. The genome will not only expand the number of phytoplasma species and provide some new information of *Ca. P.* ziziphi, but also contribute to explore its pathogenic mechanism.

## Materials and methods

### Material

*Z. jujuba* Mill. ‘Dongzao’ sensitive to *Ca. P.* ziziphi is one of the most popular cultivar. The diseased ‘Dongzao’ infected by *Ca. P.* ziziphi primarily colonizing in the phloem tissue usually exhibited typical witches’ broom symptom. Thus, the diseased trees of ‘Dongzao’ are the ideal materials to collect and isolate *Ca. P.* ziziphi. In this study, *Ca. P.* ziziphi isolated from the bark of diseased ‘Dongzao’ branches on June (2018) in Hebei, and it was then named as the strain Hebei-2018. Total DNA was extracted from the bark of diseased branches using CTAB method to diagnose the content of *Ca. P.* ziziphi.

The tissue culture of host plants is a solution for preservation and multiplication of phytoplasma. The diseased shoot tips of ‘Dongzao’ infected by *Ca. P.* ziziphi were used as explants and cultured in MS media by referring to previous study [67]. The JWB-diseased plantlets with a large number of *Ca. P.* ziziphi were subcultured and then used for different treatments. Culture conditions were as follow: the temperature was kept at 25 ± 1 °C, and the photoperiod and light intensity were 16 h/8 h and 1400–1600 lx, respectively. The JWB- diseased plantlets were treated with tetracycline (0 µg/mL, 50 µg/mL, 100 µg/mL) and cultured in media with different pH values (5, 6, 7, 8). Fifteen plantlets were treated in each treatment, and three replications (5 plantlets mixed in one replication) were sampled after 25 d of culture in different media. Above samples were used to observe morphological changes and to detect phytoplasma content and gene expression.

### Detection of ***Ca. P.*** ziziphi

*Ca. P.* ziziphi was detected by transmission electron microscopy (TEM) and 4’,6-diamidino-2-phenylindole (DAPI) staining method (2018). Meanwhile, PCR detection was also used by universal phytoplasma 16 S rRNA gene primer pair P1 and P7 [77, 78] and Thymidylate kinase (TMK) [31].

### Whole genome sequencing and annotation

The genomic DNA of *Ca. P.* ziziphi was isolated and confirmed by PCR using phytoplasma 16 S rRNA and TMK primers [31]. The genome was sequenced with Oxford Nanopore Technology by GridION X5, and the total reads were 6.8 G. 98% of raw reads assigned to bacteria and plant. The average length of Pass Reads was 16,095 bp, and the reads (length < 1 kb) were removed. The genomics sequences of jujube were removed based on the whole jujube genome sequence [79]. The completeness of assembly was evaluated using the software BUSCO v3 with a set of common phytoplasma single-copy orthologs [80]. Prodigal v2.60 [81] was used to predict open reading frames (ORFs) for the genome sequence of *Ca. P.* ziziphi. UGA was used as a stop codon, which was consistent with the ORF prediction for other phytoplasmas. tRNAscan-SE was applied to identify tRNAs [82]. RNAmmer [83] was used to predict the locations of rRNA genes.

The genes of *Ca. P.* ziziphi were named according to their homologous genes, which were identified by the OrthoMCL [84] and BLASTP [85]programs. OrthoMCL was used to identify homologous genes between the *Ca. P.* ziziphi genome and other complete phytoplasma genomes. Those genes with no homology to the other complete phytoplasma genomes were searched by BLASTP against the NCBI nr database. Afterward, the rest of the genes were presumed to be putative protein-coding genes only when they were longer than 100 amino acids or had a confidence score of more than 10 from Prodigal.

### Sequence data

Based on 16S rRNA gene analysis, eight other groups phytoplasma genomes downloaded from NCBI and *Ca. P.* ziziphi (CP091835) belonging to 16SrV-B group in this study were selected and aligned using the Multiple Alignment of Coding SEquences accounting for frame shifts and stop codons (MACSE) program. The MACSE algorithm is a useful tool for accommodating sequencing errors and other biological deviations from the coding frame [86]. Eight phytoplasmas were grouped by 16S rRNA gene as follow: *C*a. *P.* oryzae JHUK00000000.1, 16SrXI Group; *Ca. P.* mali CU469464.1, 16SrX-A Group; *Ca*. *P.* australiense, Strawberry lethal yellows phytoplasma (CPA) CP002548.1, 16SrXII-B Group; *C*a. *P.* asteris, Aster yellows witches’-broom phytoplasma CP000061.1, 16SrI-A Group; *C*a. *P.* asteris, yellows phytoplasma AP006628.2, 16SrI-B Group; *C*a. *P.* vaccinium, Vaccinium witches’-broom phytoplasma AKIN00000000.1,16SrIII-X Group; *C*a. *P.* italian, Italian clover phyllody phytoplasma AKIM00000000.1, 16SrIII-X Group; *Ca. P.* aurantifolia, Peanut witches’-broom phytoplasma AMWZ01000014.1,16SrII-B Group.

### Codon usage bias (CUB) and ENC-plot analysis

To elucidate the difference in CUB among phytoplasma species, codon usage,relative synonymous codon usage (RSCU) [87], GC content in the third position of the codon (GC3s) and effective numbers of codons (ENC) were calculated by using the program CodonW as implemented in the Galaxy server version 1.4.4 [88]. Based on the RSCU of all 59 synonymous codons, a heat map was constructed by biclustering approaches.

### Gene expression analysis

Gene expression was detected by qRT-PCR. Total RNA was extracted from JWB plantlets using TIANGEN Kit. The first-strand cDNA was synthesized in 20 µL reactions from 1 µg DNase I-treated total RNA using a reverse transcription Kit (TIANGEN, China, KR118). The primers used in this study are listed in Supplementary file-Table S5. PCR products were amplified in triplicate using Bio-Rad iQ™5 with TransStart Top Green qPCR SuperMix AQ131 (TransGen Biotech, China) in 20 µL reactions. Each reaction contained 10 µL of 2 × TransStart® Top Green qPCR SuperMix, 0.4 µL each of 10 µM primers, 8.2 µL of ddH_2_O and 1 µL of cDNA. The thermal profile for RT-qPCR was as follows: preincubation for 30 s at 95 °C, followed by 40 cycles of 5 s at 95 °C, 10 s at 55 °C, and 10 s at 72 °C. Three biological replicates were performed for each treatment. Threshold cycle values were calculated using iCycler software, and ZjACT was used as an internal control. Relative transcript levels were calculated according to the 2^–ΔCT^ method [89].

### Purification of proteins and the detection of enzyme activity

Phytoplasma SodA sequence was amplified by specific primer in JWB plantlets. And recombinant MBP-SodA proteins were purified from BL21 strain using amylose agarose. Purified MBP-tagged full-length SodA was used for SOD activity assays performed using the nitroblue tetrazolium (NBT) method. The reaction medium contained 0.1 mM NBT, 15 mM methionine, 0.003 mM EDTA, 0.1 mM riboflavin, protein sample, and a buffer to a final reaction volume of 4.2 mL, and the mixture was incubated at 25 °C for 10 min. The absorbance values were recorded at 560 nm. One unit of SOD equaled the amount of enzyme capable of inhibiting NBT photoreduction by 50%. Then, SOD activity was calculated as follows:

U/mg = (1-ΔE_sample_/ΔE_reference_)/protein amount (mL) × protein concentration (mg/mL).

### Conserved motifs analysis and phylogenetic tree construction of FtsH and SodA

The conserved motifs of FtsH and SodA proteins were detected by MEME (http://meme-suite.org/) using the following parameters: number of repetitions, any; maximum number of motifs, 20; and optimum motif widths, 6–60 amino acids [90]. MEGA 11 software and the neighbor-joining statistical method were used to construct a phylogenetic tree. The evolutionary distances were obtained using the p-distances method, and these distances were used to estimate the number of amino acid substitutions per site. The reliability of each phylogenetic tree was established by conducting 1000 bootstrap sampling iterations.

### Statistical analysis

The data was subjected to analysis of variance and tested for significant treatment differences using Duncan’s test and Student’s *t*-test (* p < 0.05, ** p < 0.01). The results are presented as mean ± standard deviation (SDs) of three replicate samples.

## Electronic supplementary material

Below is the link to the electronic supplementary material.

Supplementary Material 1

## Data Availability

The genome of *Ca. P.* ziziphi is available in GenBank databases under accession number CP091835. All data and materials are presented in the main manuscript and additional supporting files.
