# Supplementary material for: The genome of Candidatus phytoplasma ziziphi provides insights into their biological characteristics
Source: BMC Plant Biol. 2023 May 12;23:251. doi: 10.1186/s12870-023-04243-6 (PMC10176825; doi:10.1186/s12870-023-04243-6)
Supplement: Supplementary file 1 — Supplementary Material 1 [file 12870_2023_4243_MOESM1_ESM.doc]

**The Genome of *Candidatus Phytoplasma* Ziziphi Provides Insights into Their Biological Characteristics**

**Chaoling Xue^1,2 †^, Yao Zhang^1,2 †^, Hongtai Li^1,2^, Zhiguo Liu^3^, Weilin Gao^1,2^, Mengjun Liu^3^, Huibin Wang^1^, Ping Liu^3^, *, Jin Zhao^1,2,🞷^**

^1^ College of Life Science, Hebei Agricultural University, Baoding, 071000 China

^2^ Key Laboratory of Hebei Province for Plant Physiology and Molecular Pathology, Hebei Agricultural University, Baoding, 071000 China

^3^ Research Center of Chinese Jujube, Hebei Agricultural University, Baoding, 071000 China

*Corresponding authors: Jin Zhao, zhaojinbd@126.com; Ping Liu, yylp@hebau.edu.cn.

† These authors contributed equally to this work.

**Supplementary file-Fig. S1** Verification of the overlapping sequences between the newly assembled 19.825-kb sequence and the previous genome. Compared to the previous genome (Wang et al. 2018) (29), this assembly contains 19.825 kb of additional sequence, and the sequence was verified by PCR amplification. (A) PJ1 and PJ2 represent the front and back overlapping sequences between the previously reported genome (black) and the newly assembled sequence (red), respectively. The green and blue boxes are forward and reverse primers for PJ1 and PJ2, respectively. (B, C) DNAMAN was used for the alignment of PJ1 and PJ2 with the previous genome.

**Supplementary file-Fig. S2** The composition of 20 amino acids among the genomes of 9 phytoplasmas. 1- (*Ca. P.* oryzae) GCA_001578535.1; 2-Ca. P. mali GCA_000026205.1; 3- (*Ca. P.* australiense) Strawberry lethal yellows phytoplasma (CPA) GCA_00039 7185.1; 4- (*Ca. P.* asteris) Aster yellows witches’-broom phytoplasma GCA_000012225.1; 5- (*Ca. P.* asteris) Onion yellows phytoplasma GCA_000009845.1; 6 (*Ca. P.* vaccinium) Vaccinium witches’-broom phytoplasma GCA_ 000309405.1; 7- (*Ca. P.* italian) Italian clover phyllody phytoplasma GCA_000300695.1; 8- (*Ca. P.* ziziphi) JWB phytoplasma (in this study); 9- (*Ca. P.* aurantifolia) Peanut witches’-broom phytoplasma GCA_000364425.1.

**Supplementary file-Fig. S3** Conserved motifs of the SodA proteins arranged according to their phylogenetic relationships. 10 conserved motifs were shown in different colors. The motif 2 and 3 are related to Fe-dependent domain, the motif 4 is associated with Mn-dependent domain. The motif 5 is involved in Cu-Zn-dependent motif. Other motifs are unknown function.

**Supplementary file- Fig. S4** The phytoplasma content are detected using TMK primer by qPCR in plantlets under pH (5, 6, 7, 8) and tetracycline (TC_50 µg/mL_ and TC_100 µg/mL_) treatments.

**Supplementary file-Fig. S5** Conserved motifs of the FtsH proteins arranged according to their phylogenetic relationships. 10 conserved motifs were shown in different colors. The FtsH protein contained a typical AAA module of the AAA family. The AAA module contained the characteristic sequence motifs, namely Walker A (motif 2) and Walker B (motif 8) as well as the pore residues (motif 2) and the second region of homology (SRH) (motif 3) fingerprint. The ‘zinc-binding’ motif (motif 1, HEAGH) identified the protease active center. Other motifs (4, 5, 6, 7, 9, 10) were secondary structural elements that formed the protease domain.

**Supplementary file-Table S1** The general information of 28 phytoplasma genomes, The genome size, gene number, GC content and the corresponding reference.

**Supplementary file-Table S2** The top and low frequency codons in 9 phytoplasma genomes. RSCU values of all 59 synonymous codons are counted in 9 genomes, and the values are normalized. Taking the value 1 as the dividing point, the values greater than 1 are the high-frequency codon (in yellow background), and the values lower than 1 are the low-frequency codon (in blue background).

**Supplementary file-Table S3** The information analysis of 9 FtsH family genes in *Ca. P.* ziziphi, including amino acids size, the molecular weight and isoelectric point.

**Supplementary file-Table S4** The top six frequency codons in three phytoplasmas and their host plants. The top frequently codons are basically similar in phytoplasma and its host.

**Supplementary file-Table S5** The primers used in this study.


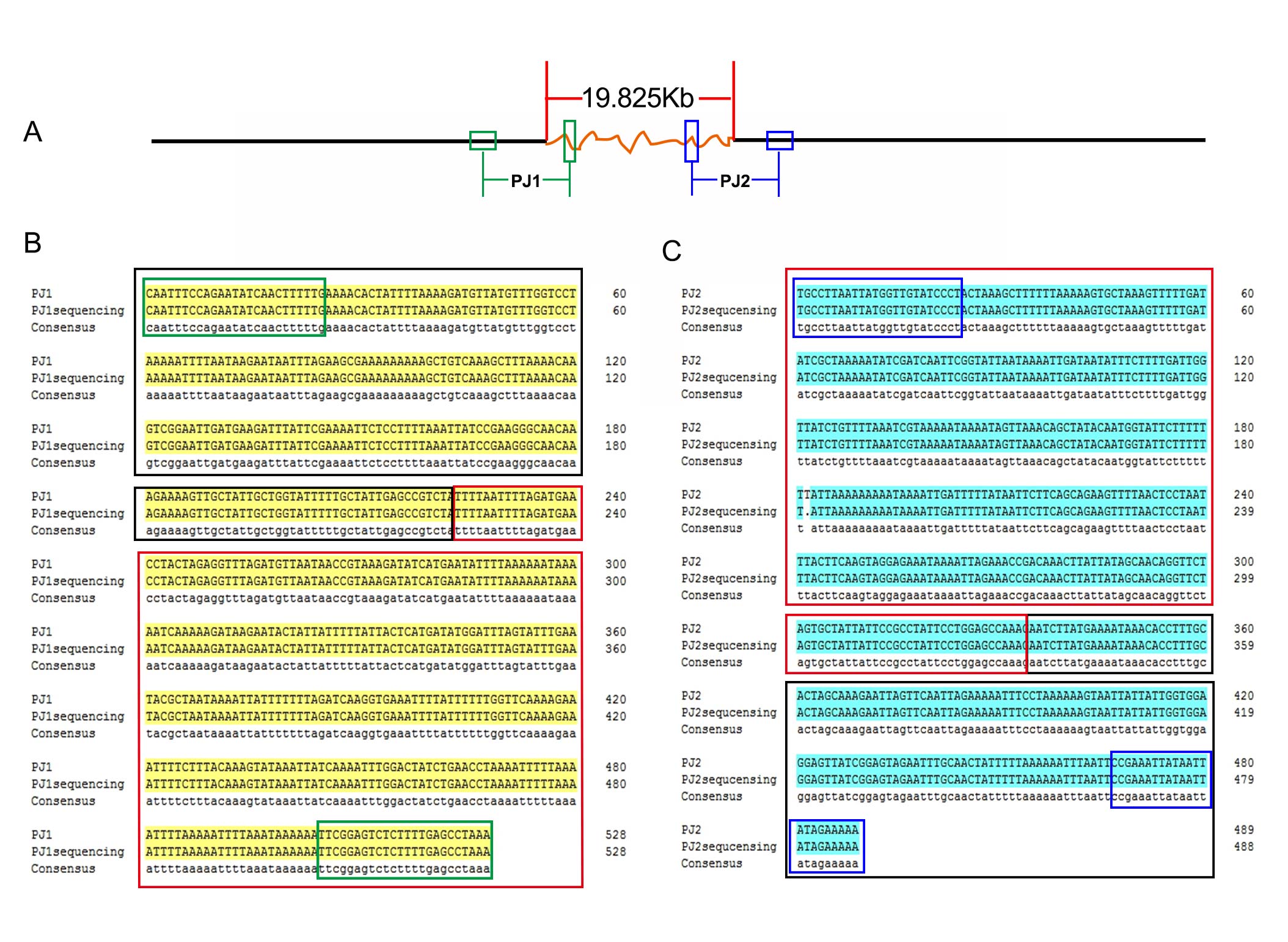


**Fig. S1** Verification of the overlapping sequences between the newly assembled 19.825-kb sequence and the previous genome. Compared to the previous genome (Wang et al. 2018) (37), this assembly contains 19.825 kb of additional sequence, and the sequence was verified by PCR amplification. (A) PJ1 and PJ2 represent the front and back overlapping sequences between the previously reported genome (black) and the newly assembled sequence (red), respectively. The green and blue boxes are forward and reverse primers for PJ1 and PJ2, respectively. (B, C) DNAMAN was used for the alignment of PJ1 and PJ2 with the previous genome.


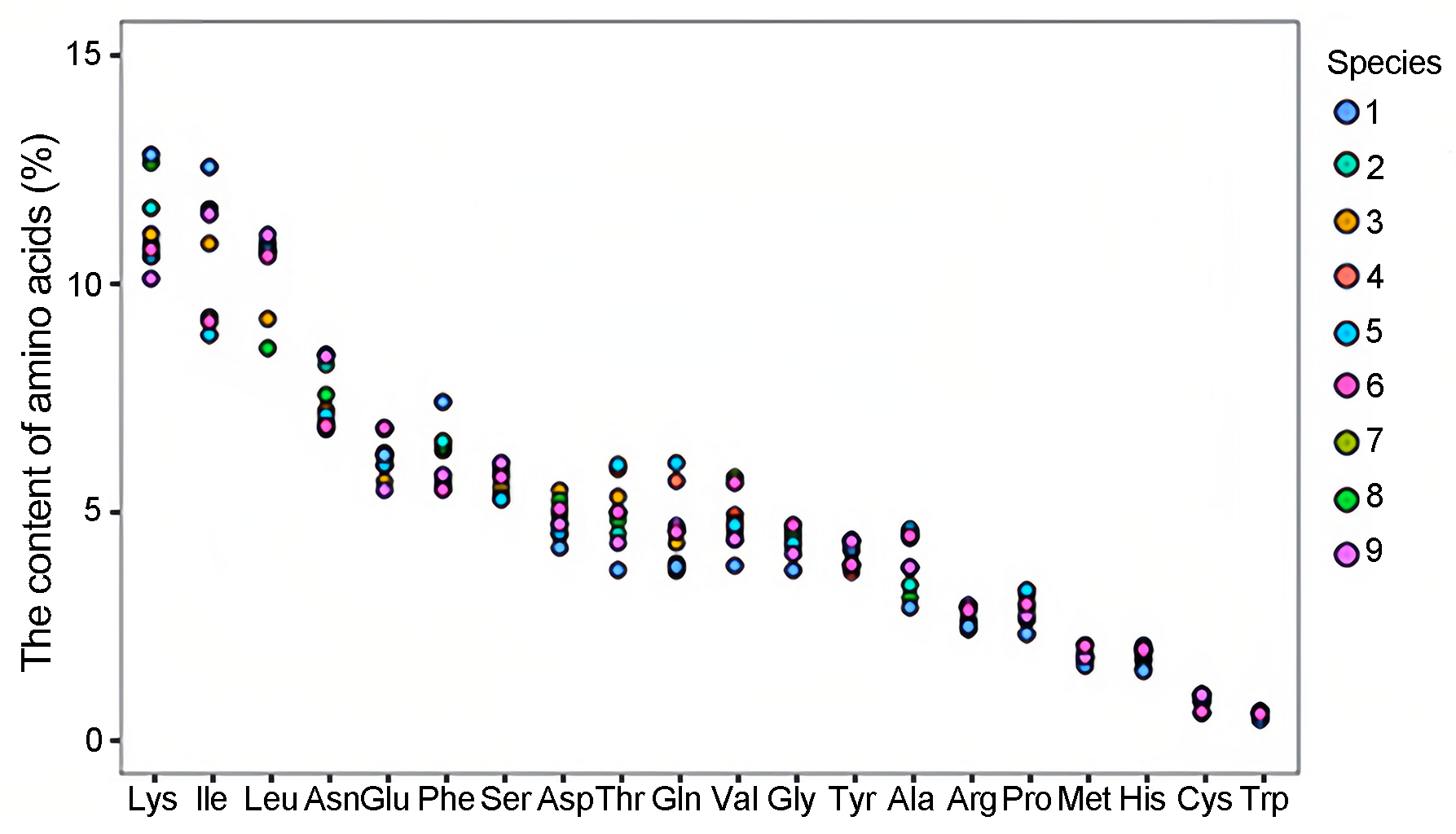


**Fig. S2** The composition of 20 amino acids among the genomes of 9 phytoplasmas. 1- (*Ca. P.* oryzae) GCA_001578535.1; 2-Ca. P. mali GCA_000026205.1; 3- (*Ca. P.* australiense) Strawberry lethal yellows phytoplasma (CPA) GCA_00039 7185.1; 4- (*Ca. P.* asteris) Aster yellows witches’-broom phytoplasma GCA_000012225.1; 5- (*Ca. P.* asteris) Onion yellows phytoplasma GCA_000009845.1; 6 (*Ca. P.* vaccinium) Vaccinium witches’-broom phytoplasma GCA_ 000309405.1; 7- (*Ca. P.* italian) Italian clover phyllody phytoplasma GCA_000300695.1; 8- (*Ca. P.* ziziphi) JWB phytoplasma (in this study); 9- (*Ca. P.* aurantifolia) Peanut witches’-broom phytoplasma GCA_000364425.1.


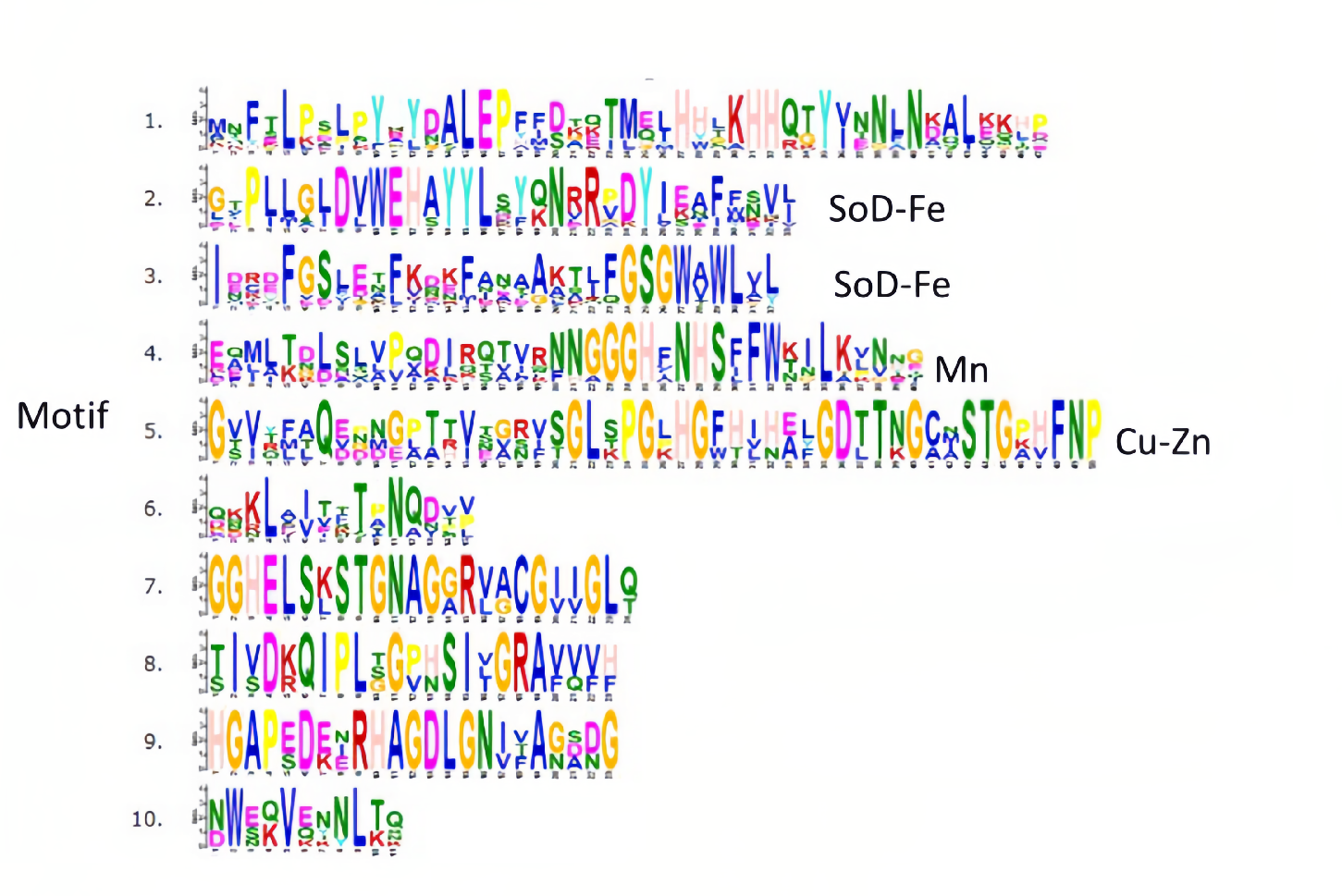


**Fig. S3** Conserved motifs of the SodA proteins arranged according to their phylogenetic relationships. 10 conserved motifs were shown in different colors. The motif 2 and 3 are related to Fe-dependent domain, the motif 4 is associated with Mn-dependent domain. The motif 5 is involved in Cu-Zn-dependent motif. Other motifs are unknown function.


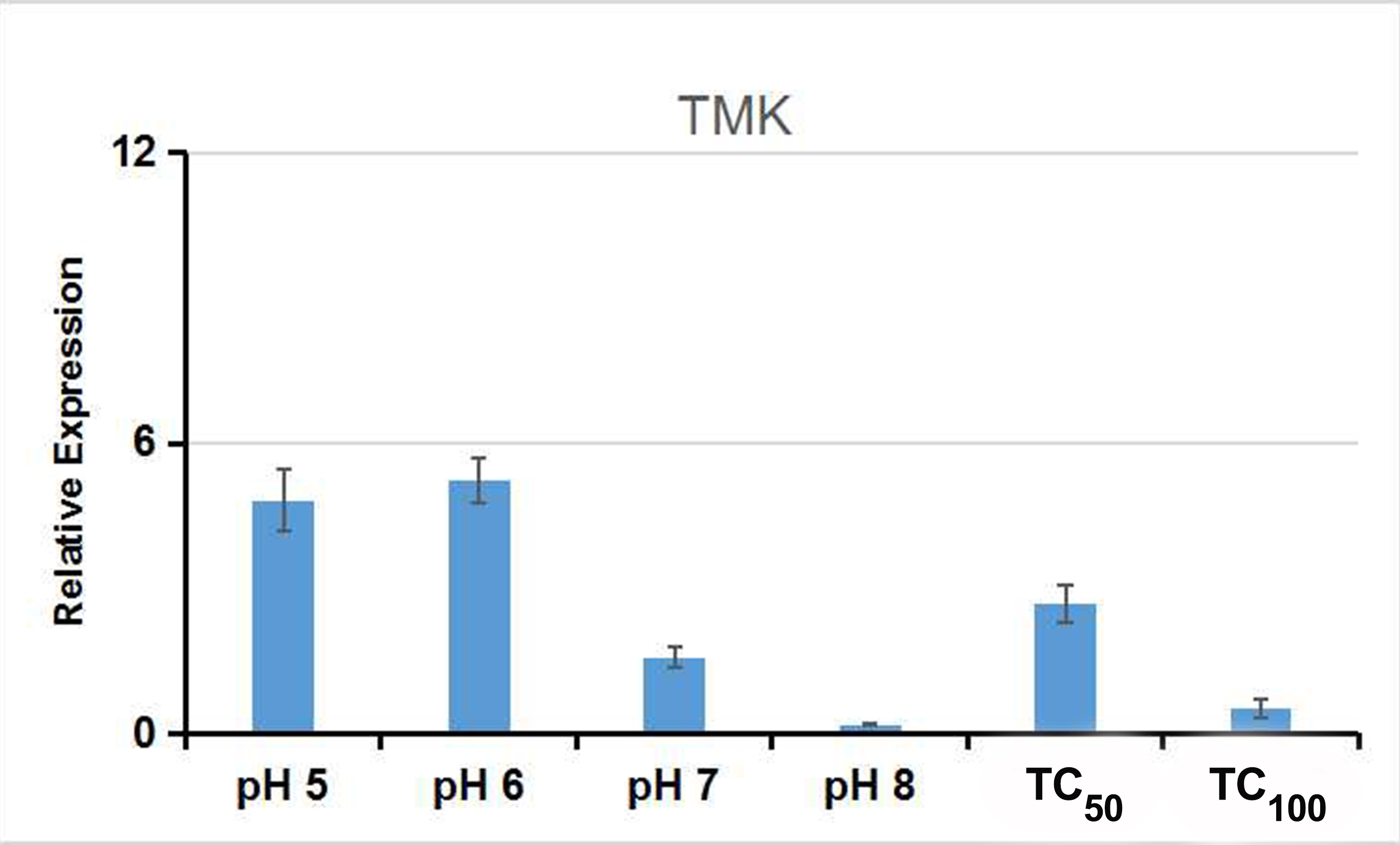


**Fig. S4** The phytoplasma content are detected using TMK primer by qPCR in plantlets under pH (5, 6, 7, 8) and tetracycline (TC_50 µg/mL_ and TC_100 µg/mL_) treatments.


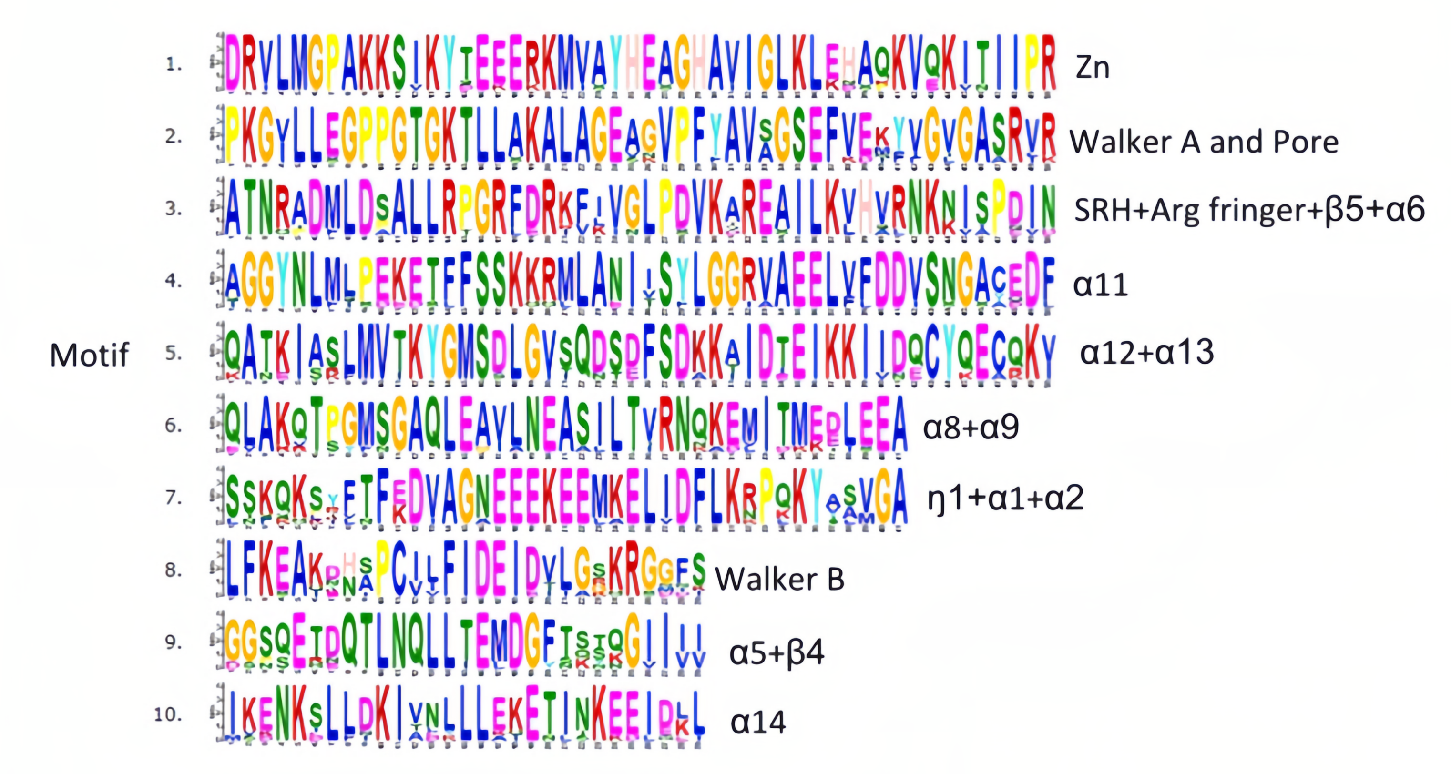


**Fig. S5** Conserved motifs of the FtsH proteins arranged according to their phylogenetic relationships. 10 conserved motifs were shown in different colors. The FtsH protein contained a typical AAA module of the AAA family. The AAA module contained the characteristic sequence motifs, namely Walker A (motif 2) and Walker B (motif 8) as well as the pore residues (motif 2) and the second region of homology (SRH) (motif 3) fingerprint. The ‘zinc-binding’ motif (motif 1, HEAGH) identified the protease active center. Other motifs (4, 5, 6, 7, 9, 10) were secondary structural elements that formed the protease domain.

| **Table S1 The general information of 28 phytoplasma genomes** | | | | | |  |
| --- | --- | --- | --- | --- | --- | --- |
| **Name** | **Genome size(bp)** | **No. of genes** | **GC content %** | **Group** | **Reference** | |
|
| AYWB | 860,631 | 446 | 28.0 | 16SrI-A | Bai X, et al. 2006^[5]^ | |
| *Candidatus Phytoplasma* australiense | 879,959 | 684 | 27.4 | 16SrXII | Tran-Nguyen LT, et al 2008^[6]^ | |
| *Candidatus Phytoplasma* mali | 601,943 | 479 | 21.4 | 16SrX | Kube M, et al.2008^[7]^ | |
| *Candidatus Phytoplasma* pruni | 598,511 | 550 | 27.2 | 16SrIIIA | Lee IM, et al. 2015^[11]^ | |
| OY-M | 853,092 | 749 | 27.8 | 16SrI-B | Oshima K, et al.2004^[4]^ | |
| PnWB NTU2011 | 562,473 | 421 | 24.3 | 16SrII | Chung WC, et al. 2013^[10]^ | |
| Wheat blue dwarf phytoplasma | 610,000 | 471 | 27.1 | 16SrI | Chen W, et al. 2014^[9]^ | |
| *Candidatus Phytoplasma* vitis | 528,000 | 482 | 21.1 | 16SrV | Carle P et al. 2011^[12]^ | |
| maize bushy stunt phytoplasma MBSP | 576,118 | 531 | 26.2 | 16SrIB | Orlovskis Z, et al.2017^[13]^ | |
| *Candidatus Phytoplasma* oryzae | 533,195 | 462 | 19.30 | 16SrXI | Fischer A, et al. 2016^[14]^ | |
| Vaccinium Witches’ Broom phytoplasma | 647,754 | 677 | 27.4 | 16SrIII-F | Saccardo F, et al. 2012^[8]^ | |
| Milkweed Yellows phytoplasma | 583,806 | 650 | 27.5 | 16SrIII-F | Saccardo F, et al. 2012^[8]^ | |
| Italian Clover Phyllody phytoplasma | 597,245 | 565 | 27.1 | 16SrIII-B | Saccardo F, et al. 2012^[8]^ | |
| Poinsettia branch-inducing phytoplasma | 631,440 | 654 | 27.3 | 16SrIII-H | Saccardo F, et al. 2012^[8]^ | |
| Echinacea purpurea' witches'-broom phytoplasma | 545,427 | 433 | 23.9 | 16SrII-A | Chang SH, et al. 2015^[15]^ | |
| *Candidatus Phytoplasma* phoenicium | 406,850 | 333 | 26.0 | 16SrIX-B | Quaglino F, et al. 2015^[16]^ | |
| *Candidatus Phytoplasma* solani | 821,322 | 709 | 28.3 | 16SrXII | Music MS, et al. 2019^[17]^ | |
| *Candidatus Phytoplasma* sacchari | 505,173 | 404 | 19.86 | 16SrXI | Kirdat K, et al. 2020^[18]^ | |
| 'Cynodon dactylon' phytoplasma | 483,935 | 433 | 20.5 | 16SrXIV | Kirdat K, et al. 2020^[18]^ | |
| *Candidatus Phytoplasma* pini | 474,136 | 401 | 22.2 | 16SrXXI-B | Cai W, et al. 2020^[19]^ | |
| Periwinkle Leaf Yellowing (PLY) Phytoplasmas | 824,596 | 775 | 27.6 | 16SrI-B | Cho ST, et al. 2019^[21]^ | |
| *Candidatus Phytoplasma* meliae | 751,949 | 657 | 27.3 | 16SrXIII-G | Fernández FD, et al. 2016^[23]^ | |
| *Candidatus Phytoplasma* luffae | 769,143 | 720 | 23.3 | 16SrVIII-A | Davis RE, et al. 2017^[22]^ | |
| Rapeseed phyllody phytoplasma | 829,546 | 753 | 27.7 | 16SrI-B | Cho ST, et al. 2020^[21]^ | |
| *Candidatus Phytoplasma* aurantifolia | 474,669 | 386 | 23.9 | 16SrII | Al-Ghaithi AG, et al. 2018^[24]^ | |
| New Jersey aster yellows phytoplasma | 652,092 | 733 | 27.1 | 16SrI-A | Sparks ME, et al. 2018^[25]^ | |
| Rice orange leaf phytoplasma | 599,264 | 647 | 28.2 | 16 SrI | Zhu Y, et al. 2017^[26]^ | |
| Chrysanthemum yellows phytoplasma | 659,699 | 573 | 28.3 | 16SrI | Pacifico D, et al. 2015^[27]^ | |

| **Table S2 The top and low frequency codons in 9 phytoplasmas** | | | | | | | | | |
| --- | --- | --- | --- | --- | --- | --- | --- | --- | --- |
|  | ***Ca. P.* oryzae** | ***Ca. P.* mali** | ***Ca. P.* australiense**  **CPA** | ***Ca. P.* asteris**  **Aster** | ***Ca. P.* asteris**  **Onion** | ***Ca. P.* vaccinium** | ***Ca. P.* italian** | ***Ca. P.* ziziphi** | ***Ca. P.* aurantifolia**  **PnWB** |
| **Top frequency codons** | TTA(4.62)Leu | TTA(4.66)Leu | TTA(3.69)Leu | TTA(3.41)Leu | TTA(3.42)Leu | TTA(4.25)Leu | TTA(4.24)Leu | TTA(4.46)Leu | TTA(4.43)Leu |
|  | AGA(4.01)Arg | AGA(3.18)Arg | AGA(2.61)Arg | AGA(2.74)Arg | AGA(2.68)Arg | AGA(3.14)Arg | AGA(3.16)Arg | AGA(3.41)Arg | GCT(2.55)Ala |
|  | TCT(2.84)Ser | GTT(2.58)Val | GTT(2.53)Val | GTT(2.19)Val | CCT(2.14)Pro | TCT(2.66)Ser | TCT(2.63)Ser | TCT(2.61)Ser | TCT(2.51)Ser |
|  | GCT(2.76)Ala | GCT(2.46)Ala | CCT(2.08)Pro | CCT(2.16)Pro | GTT(2.13)Val | GCT(2.47)Ala | CCT(2.49)Pro | CCT(2.39)Pro | CCT(2.44)Pro |
|  | CCT(2.65)Pro | TCT(2.41)Ser | TCT(2.03)Ser | TCT(2.12)Ser | TCT(2.03)Ser | CCT(2.44)Pro | GCT(2.45)Ala | GTT(2.37)Val | AGA(2.32)Arg |
|  | GTT(2.47)Val | ACT(2.23)Thr | GCT(1.94)Ala | ACT(2.01)Thr | ACT(2.02)Thr | GTT(2.19)Val | ACT(2.19)Thr | GCT(2.35)Ala | GTT(2.31)Val |
|  |  |  |  |  |  |  | GTT(2.19)Val |  |  |
| **Low frequency codons** | CCC(0.11)Pro | AAG(0.12)Lys | ACG(0.16)Thr | ACG(0.11)Thr | AAG(0.12)Lys | GAG(0.16)Glu | GAG(0.17)Glu | GAG(0.13)Glu | AAG(0.15)Lys |
|  | CAG(0.11)Gln | GTC(0.12)Val | AAG(0.15)Lys | AAG(0.11)Lys | GAG(0.12)Glu | AAG(0.14)Lys | AAG(0.15)Lys | AAG(0.12)Lys | GAC(0.15)Asp |
|  | AAG(0.11)Lys | GAG(0.12)Glu | GAG(0.14)Glu | GAG(0.11)Glu | ACG(0.1)Thr | CTG(0.09)Leu | CTG(0.09)Leu | CTC(0.08)Leu | CAG(0.14)Gln |
|  | GAC(0.11)Glu | CAG(0.10)Gln | CGG(0.10)Arg | CTG(0.07)Leu | CTG(0.08)Leu | CTC(0.08)Leu | CAG(0.08)Gln | CAG(0.08)Gln | CGG(0.13)Arg |
|  | GAG(0.11)Glu | CGG(0.10)Arg | CAG(0.08)Gln | CAG(0.05)Gln | CGG(0.07)Arg | CAG(0.08)Gln | CTC(0.07)Leu | CGG(0.05)Arg | CTC(0.06)Leu |
|  | CGC(0.06)Arg | CTG(0.05)Leu | CTG(0.07)Leu | CGG(0.05)Arg | CAG(0.05)Gln | CGG(0.05)Arg | CGG(0.04)Arg | CTG(0.04)Leu | CTG(0.05)Leu |
|  | CTC(0.04)Leu | CTC(0.02)Leu |  |  |  |  |  |  |  |
|  | CTG(0.04)Leu |  |  |  |  |  |  |  |  |
|  | CGG(0.03)Arg |  |  |  |  |  |  |  |  |

| **Table S3**  **The information analysis of 9 FtsH family genes in *Ca. P.* ziziphi** | | | |
| --- | --- | --- | --- |
| **Gene name** | **Size (aa)** | **MW(KD)** | **PI** |
| FtsH1 | 446 | 49.23 | 6.697 |
| FtsH2 | 593 | 67.20 | 7.211 |
| FtsH3 | 532 | 59.91 | 8.773 |
| FtsH4 | 440 | 48.71 | 7.495 |
| FtsH5 | 550 | 62.09 | 8.482 |
| FtsH6 | 583 | 66.08 | 9.448 |
| FtsH7 | 453 | 50.26 | 8.371 |
| FtsH8 | 596 | 68.25 | 9.996 |
| FtsH9 | 665 | 75.47 | 6.895 |

| **Table S4 The top six frequency codons in three phytoplasmas and their host plants** | | | | | |
| --- | --- | --- | --- | --- | --- |
| ***Ziziphus jujuba*** | ***Ca. P.*** ziziphi | ***Malus domestica*** | ***Ca. P.*** mali | ***Fragaria vesca*** | **CPA** |
| AGA | AGA | AGA | AGA | AGA | AGA |
| GTT | GTT | GTT | GTT | GTT | GTT |
| GCT | GCT | GCT | GCT | GCT | GCT |
| TCT | TCT | TCT | TCT | TCT | TCT |
| TTG | TTA | TTG | TTA | TTG | TTA |
| CTT | CCT | AGG | CCT | AGG | CCT |

| **Table S5 The primers used in this study** | |
| --- | --- |
| **Primer Name** | **Sequence (5' to 3')** |
| FtsH1-S | GGCTTACCATGAAGCTGGACA |
| FtsH1-F | TCTTCAGCTACTCTACCGCCTA |
| FtsH2-S | CGGAGCTACTAATAGAGCGGA |
| FtsH2-F | TTGAGCGCCACTCATACCTG |
| FtsH3-S | TTTAGGCGGTAGAGTAGCTGAA |
| FtsH3-F | AACACCTAAATCACTCATTCCAT |
| FtsH4-S | AGTGGCGCTCAATTAGAAGCA |
| FtsH4-F | CAGCTTCATGGTAAGCCACC |
| FtsH5-S | GCTTAGCTCCGCAAATGGTT |
| FtsH5-F | GATTTGGTCCTCGTGGTTGG |
| FtsH6-S | TTAGGCGGTAGAGTAGCTGA |
| FtsH6-F | GGCATTCTTGATAACATTCGT |
| FtsH8-S | TCCAAGAGGTTCTATCGGTGG |
| FtsH8-F | TCTTCGGCTACTATACCGCC |
| FtsH9-S | ACACTTGCTCGCAAAAGAGG |
| FtsH9-F | CGACGTTCTAACCTTCCTGGT |
| Ftsy-S | GCTATGATTGGGCAGAACGGA |
| Ftsy-F | TCCGCCTTTAGCAATTCCATC |
| ffh-S | AGCCGATGCTCTTTTAGGTCA |
| ffh-F | AGAAGCCATGCGTTCTGGAT |
| SodA-S | TGGAAGTGGTTGGACATGGTT |
| SodA-F | TCTGGACGGCGGTTTTGATAA |
| SecA-S | GGCAGGTCGTGGTACTGAT |
| SecA-F | TAACCAGGGTCTCCTTGACG |
| SecY-S | AGATGTCGCGGAACATTTATCA |
| SecY-F | CTAGTCCCGCCAAACGGAA |
| YidC-S | GTGTATCGGAGAAGGATGGTT |
| YidC-F | TTTCTGGTTGAGCCAAACTC |
